# Discovery of novel glycosylation methods using Bayesian optimization: lithium salt directed stereoselective glycosylations†

**DOI:** 10.1039/d5sc03244j

**Published:** 2025-07-08

**Authors:** Natasha Videcrantz Faurschou, Christian Marcus Pedersen

**Affiliations:** a Department of Chemistry, University of Copenhagen Universitetsparken 5 2100 Copenhagen Ø Denmark cmp@chem.ku.dk

## Abstract

In recent years, Bayesian optimization has gained increasing interest as a tool for reaction optimization. Here we use Bayesian optimization in a reaction discovery fashion by treating the glycosylation reaction class as a black box function. This provides access to new areas of the glycosylation reaction space and leads to the discovery of novel stereoselective glycosylation methodologies, where stereoselectivity can be directed by the addition of lithium salts in interplay with other reaction conditions. Black box functions are inherently difficult to interpret, but we show how partial dependence plots can be used to infer trends from the obtained data in a similar fashion to the commonly used one-variable-at-time approach.

## Abstract 2

Bayesian optimization enables the discovery of novel stereoselective glycosylations.

## Graphical abstract

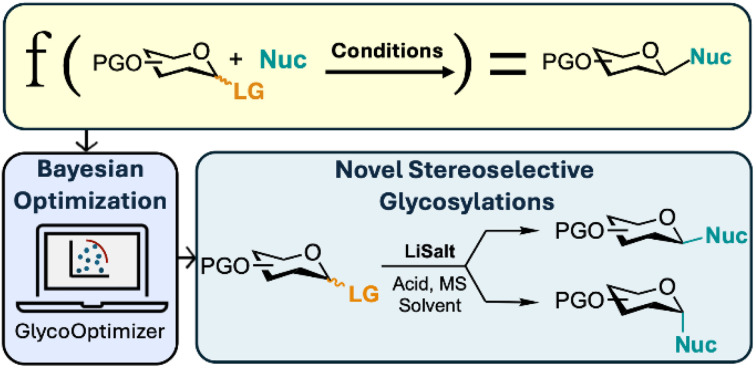

## Introduction

**Fig. 1:**
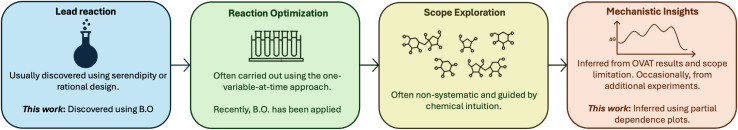
A typical workflow for reaction discovery. First, a lead reaction is discovered, and then the reaction conditions are optimized to maximize yield, selectivity, *etc.* Next, the reaction scope for the methodology is explored, and the mechanism is rationalized in hindsight.

Reaction discovery and the development of new synthetic methodologies are core topics within organic chemistry. A typical academic workflow for reaction discovery is depicted in Fig. 1. The lead reaction is often found through sheer serendipity or hypotheses based on chemical rationalization. More recently, developments in the field of analytic chemistry, automatization, and artificial intelligence have allowed high-throughput experimentation (HTE) and machine learning (ML) to aid in the search for novel reactivity.^1–6^ Despite a constant broadening of our understanding of reaction mechanisms and the influence of various reaction conditions on these, most proposed mechanisms are highly simplified. This makes the rationalization and prediction of undiscovered reactivity challenging and most often mechanisms are therefore rationalized retrospectively. When a lead reaction is discovered it is optimized for yield, selectivity, or other desirable parameters. In academia, the most common strategy for reaction optimization is the one-variable-at-a-time (OVAT) approach, where statistical strategies like design of experiment (DOE) are more widespread in industry.^7^ Besides assisting in finding optimal reaction conditions, the OVAT approach is useful for understanding the influence of individual reaction parameters. Since only one reaction parameter is varied at a time, it is easy to analyze trends and try to give them chemical meaning *e.g.* relating a change in the outcome when changing the solvent to the polarity of the solvent. Recently, Bayesian optimization (BO) has been successfully applied for the optimization of multiple reactions.^7–13^ Once the optimal reaction conditions are identified, the scope of the established methodology is explored by testing different combinations of substrates. Lastly, the mechanism is often discussed based on the findings from the reaction optimization and scope exploration, and in some cases, additional experiments will be carried out to gain a deeper mechanistic insight.

New tools for discovering lead reactions for novel methodologies are desirable, especially in cases where rational design can be difficult due to complex mechanisms. An example of a reaction where our understanding of the fundamental reaction mechanism limits the rational design of new methodologies is the glycosylation reaction. One of the main challenges when designing glycosylations is controlling the anomeric selectivity, which is highly important for biological function.^14–16^

**Fig. 2:**
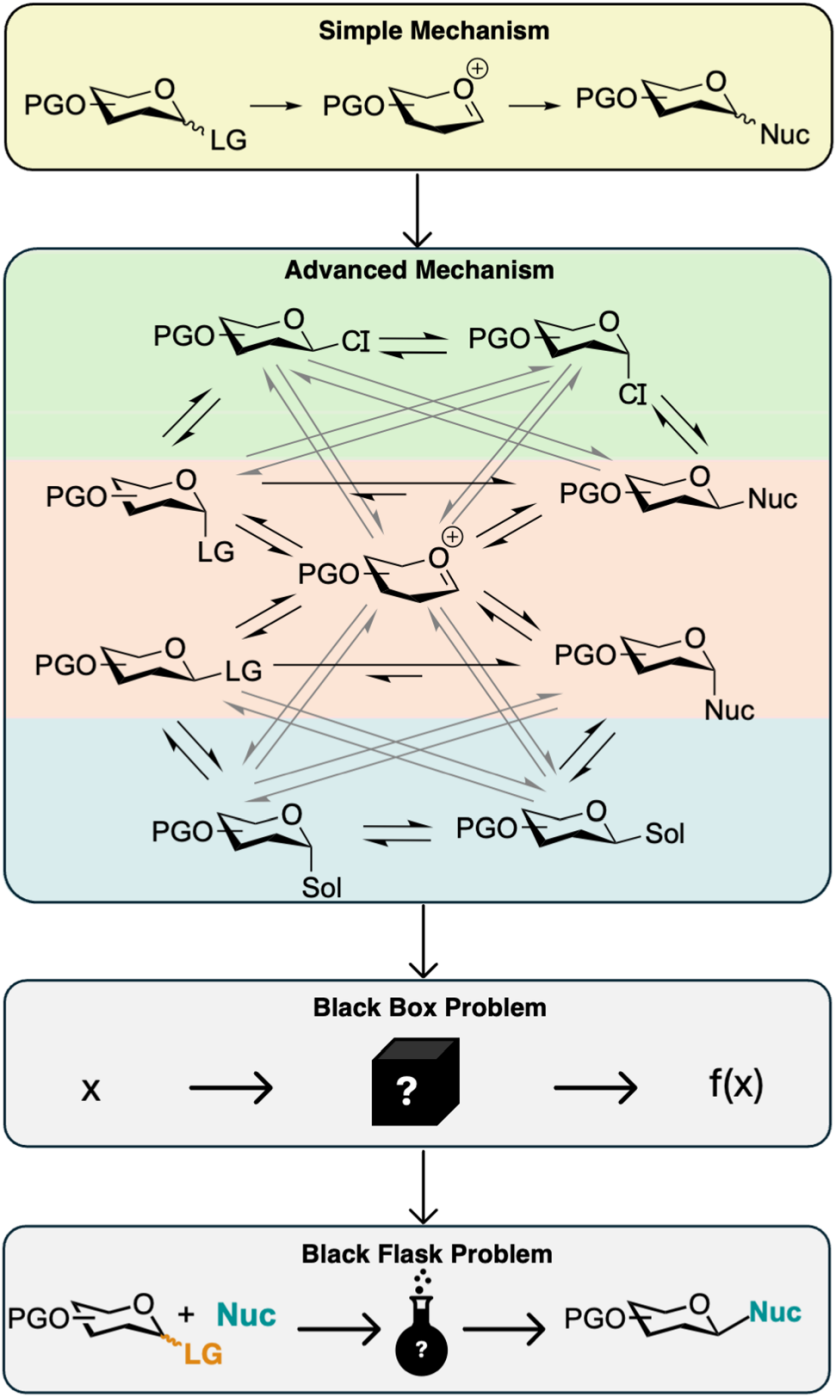
Top: A simple commonly accepted mechanism for the glycosylation reaction displayed. Below is a more advanced mechanism depicted, which more closely resembles the true reaction path with multiple species involved and all in dynamic equilibria. Both solvent and counter ions (CIs) can participate in the formation of intermediates. However, it is still a simplification and understanding the relationship between these equilibria is extremely difficult. The glycosylation reaction can therefore be viewed as a black box problem, or more aptly, a black flask problem.

Mechanistic understanding of the glycosylations reaction can help predict and guide the anomeric selectivity, and multiple mechanistic studies of glycosylations have been conducted.^17–20^ In the simplest scenario, the glycosylation reaction is considered an S_N_1-reaction with formation of a relatively stable oxocarbenium ion (Fig. 2 top). However, it is well-known that this is a very simplified view of the reaction mechanism, and a lot of work has gone into understanding the influence of different reaction conditions and substrate effects.^21^ Much work has also gone into trying to identify intermediates, both covalent adducts and ion pairs, formed during the reaction.^17,18,22–25^

Despite many detailed investigations, the general understanding of the glycosylation reaction is limited, and advanced mechanistic scenarios are only described for specific activator/leaving group systems.^17–20^ As seen from Fig. 2 (advanced mechanism), the mechanism gets increasingly complicated when including more possible intermediates. In red is highlighted the “classic” glycosylation mechanism, where the glycosylation reaction is viewed as a nucleophilic substituent reaction proceeding through either a more S_N_1-like mechanism, a more S_N_2-like mechanism, or both in competition. Figuring out where on the S_N_1/S_N_2-spectrum a specific glycosylation belongs is in itself challenging, and this will be dependent on both the substrates and conditions.^21,26^ In green, the formation of intermediates through reaction with a counter ion of the activator is also considered, here drawn as covalent adducts, but ion pairs are also known to be involved. Examples of such intermediates include glycosyl chlorides^27^ and glycosyl triflates.^18^ In blue, intermediates formed by reaction with the solvent are included, further complicating the mechanism. The advanced mechanism shown in Fig. 1 is still a simplified picture and does for instance not consider pathways with anchimeric assistance or contact ion pairs. To rationalize the outcome of glycosylations we would have to determine the relationship between all of these equilibria, but as of now, we do not have any way for assessing their individual contribution and co-dependence. Thus, a holistic understanding of the glycosylation mechanism might be impossible given our current tools. The glycosylation reaction can therefore be described as a black box/flask function (Fig. 2), that is, if we put in **x** (substrates and reaction condition) we get an outcome, *f*(**x**) (yield and stereoselectivity), but our understanding of how **x** becomes *f*(**x**) is highly limited. We therefore chose to treat the glycosylation reaction and its mechanism as a “black flask” problem and carry out a multiobjective optimization of the glycosylation reaction class by utilizing BO to try to discover new stereoselective glycosylation methodologies. As mentioned earlier, BO has in recent years been extensively applied to the reaction optimization part of the reaction discovery pipeline and also recently using a more discovery-driven approach for designing new materials^28–30^ and new catalysts.^31–34^ BO efficiently explores complex, high-dimensional spaces with limited and noisy data, making it an ideal strategy for advanced chemical systems. We envisioned BO could help in designing new glycosylation methodologies, thus shifting the application of BO from pure reaction optimization towards lead discovery in Fig. 2 by identifying new glycosylation strategies. Additionally, we show how trends for specific reaction parameters can be inferred and analyzed from the BO campaign data in a similar fashion to the analyses of OVAT data. This is done using partial dependence plots, thereby overcoming one of the obstacles of using BO compared to OVAT.

## Results and discussion

### Design of experimental setup

**Fig. 3:**
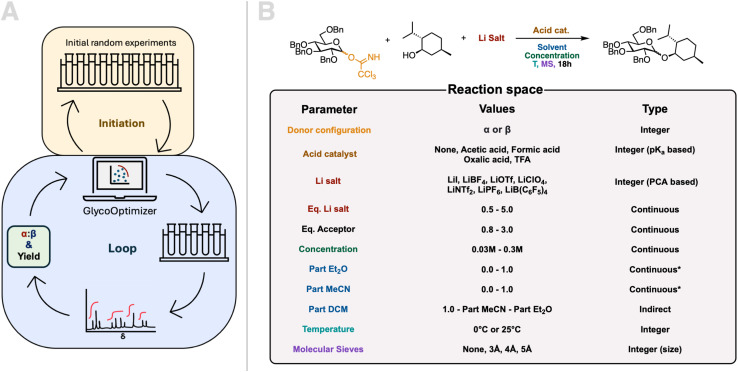
(A) An illustration of the experimental optimization loop and initiation. The first batch consists of 10 randomly suggested experiments, and the results are fed to the optimizer which proposes a batch of five new experiments using a Bayesian optimization algorithm. The results (anomeric selectivity and yield) are obtained by NMR analysis. (B) Illustration of model reaction and reaction space available for the GlycoOptimizer. The values and the representation of the reaction parameters are indicated.

The reaction discovery campaigns were run using a human-in-the-loop setup. A modified version of the Bayesian optimization algorithm ProcessOptimizer^35–37^ was used to suggest the experiments. This algorithm has previously been used for reaction optimization^8^ and can take both continuous and discrete variables as input. The algorithm has been modified to incorporate variable constraints for multiobjective optimizations. As the GlycoOptimizer is inherently a minimizer, the objectives have been modified accordingly *i.e.* 100 – objective in percentage. This modified algorithm will in the following be referred to as the GlycoOptimizer. Experiments and workup were carried out by hand (details can be found in ESI Section 2.3†). The objectives, *i.e.* yield and anomeric selectivity, were evaluated by NMR analysis using an internal standard. The experimental setup is illustrated in Fig. 3A. The campaign was initiated by a batch of 10 random experiments suggested by the GlycoOptimizer. The results from these were fed to the GlycoOptimizer which then proposed a batch of 5 new experiments. The experiments were proposed either using an estimated Pareto Front^38^ (exploitation) or Steinerberger-sampling^39^ (exploration), with a chance of Steinerberger-sampling being used of 25%. The results inferred from NMR for the proposed experiments were fed back to the optimizer, which suggested 5 new experiments and so forth. It should be noted that due to measurement limitations, the conditions under which the experiments were carried out were not always an exact match for the conditions proposed by the GlycoOptimizer with regards to equivalents and concentration and the conditions being fed back to the optimizer were the actual conditions the experiments had been carried out under.

### Design of model reaction and reaction space

Fig. 3B shows the model reaction and reaction space. The reactants are perbenzylated glucosyl trichloroacetimidate (TCA) and l-menthol as the glycosyl donor and glycosyl acceptor, respectively. A perbenzylated glycosyl donor was chosen to avoid neighboring group participation (NGP) and remote participation as we were interested in developing a method where the stereoselectivity is reagent-controlled rather than substrate-dependent. The glycosyl donor was chosen to be a TCA as TCAs are easy and cheap to synthesize from the hemiacetal, trichloroacetonitrile, and base catalyst.^40,41^ Additionally they are relatively stable and each anomer can be selectively synthesized by the choice of base.^40^l-Menthol is a commonly used glycosyl acceptor in model glycosylation reactions,^42–44^ as it shares similarities with free secondary alcohol on a monosaccharide.

When selecting the reaction space, we aimed to include as many parameters as possible that influence glycosylation outcomes. In total 11 parameters were chosen as shown in Fig. 3B. All the parameters are either represented as integers or continuous variables.

The TCA-donor configuration, α or β, was included to take into account that glycosylations can be stereospecific.^45–47^ TCAs are most commonly activated by acid catalysis, often using strong acids, but milder acids have also been shown to be sufficient.^45,48,49^ We chose to include acids with p*K*_a_s in the range of 4.8 to 0.2 represented as integers assigned according to acidity, and also with the option of no acid. We avoided stronger acids as we wanted the conditions to be as mild as possible, improving the possibility for upscale and reproducibility by non-experts.

It has been shown that the counterion of the acid can play a role in the outcome of glycosylations with regard to yield and selectivity.^17,50,51^ To mimick the counterion effect this a lithium salt was added, and the salts were assigned an integer according to a principle component analysis (PCA). Details on the PCA can be found in ESI (Section 3).† Both concentration,^42,52^ temperature,^42,53^ and solvent^42,51,54^ are also known to be important and were included as input parameters. The most well-known solvent effects within carbohydrate chemistry are the ether effect^54^ and the nitrile effect.^54^ Thus we chose a three-part solvent system to take these into account, with both part Et_2_O and part MeCN being input variables with the sum of these constrained to equal to or less than 1. If the sum is less than one, the remaining part solvent will be DCM, thus part DCM is included as an indirect variable. Temperature is included in the reaction space as a discrete variable and not a continuous variable since each temperature requires a separate reaction station. The reactions were either carried out at 25 °C or in a fridge with a temperature of 0 °C, which are the most common reaction temperatures.^49^ The presence and the size of molecular sieves have also been shown to affect the outcome of glycosylations,^51^ and were therefore also added as an input parameter as integers according to size.

### Yield and stereoselectivity optimization campaigns

**Fig. 4:**
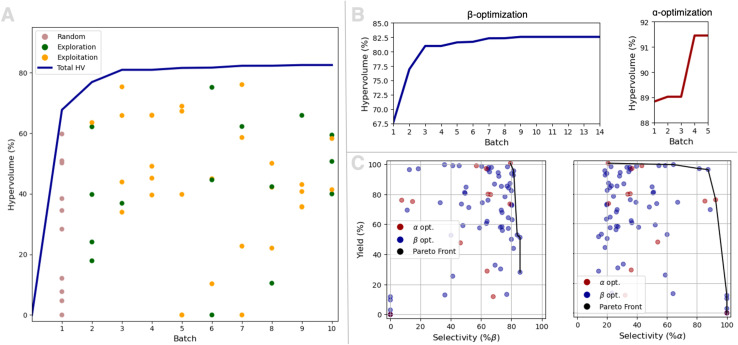
(A) Results from yield and β-selectivity optimization campaign. The first batch consists of 10 random experiments, and the other batches consist of 5 experiments suggested by the GlycoOptimizer based on the previous experiments either by estimating the Pareto Front (exploitation) or Steinberger sampling (exploration). The blue line shows the total hypervolume for all experiments and the dots indicate the hypervolume contribution for each experiment. (B) Left: convergence plot for yield and β-selectivity optimization with total hypervolume after each batch. Right: convergence plot for yield and α-selectivity optimization with total hypervolume after each batch and the first batch being all the experiments from the 10 first batches from the β-selectivity campaign. (C) The objectives for both campaigns are plotted against each other with the estimated Pareto front highlighted.

The first campaign aimed to optimize the yield and β-selectivity of the glycosylation through multiobjective optimization. In total 10 loops were carried out including the initiation batch with 10 random experiments. The results from each batch are shown in Fig. 4A as the total hypervolume and each experiment's hypervolume contribution. Hypervolumes are a way of evaluating multiobjective optimizations,^55^ and a hypervolume contribution of 100% corresponds to 100% yield and 100% stereoselectivity.

It is seen from Fig. 4A that after batch 3 only minor improvements to the total hypervolume are observed. In general, the experiments selected using the exploitative algorithm seem to have the highest hypervolume contributions, while the experiments selected using the more explorative algorithm are more scattered.

After the first 10 batches, it seemed that the optimization was near convergence, but we still envisioned that minor improvements might be possible. However, we were also interested in running a yield and α-selectivity optimization campaign, to see if we could also find a stereoselective procedure for obtaining the more challenging 1,2-*cis*-glycoside. We, therefore, decided to run a dual optimization campaign still with batches of 5 experiments, but with only two experiments proposed by the yield and β-selectivity optimizer. The last three experiments were proposed by a new yield and α-selectivity optimizer, and after each loop, the results from all 5 experiments were fed to both optimizers. The yield and α-selectivity optimization was initiated using all the data obtained from the first campaign. All 5 experiments in each batch were chosen using Pareto front sampling. From Fig. 4B it is seen that the total hypervolume for the yield and β-selectivity does not improve after the initial 10 batches *i.e.* does not improve during the second dual campaign.

As seen from Fig. 4B the total hypervolume for the yield and α-selectivity optimization is ∼89% at the beginning of the optimization, that is only with the experiments from the initial yield and β-selectivity campaign. The dual optimization campaign is terminated once no improvement is observed for yield and β-selectivity nor yield and α-selectivity.

In Fig. 4C are the yield of all glycosylation plotted against the β-selectivity (left) and the α-selectivity (right), and the estimated Pareto fronts are highlighted. For the β-selective glycosylations, it seems that the limiting objective is the stereoselectivity, whereas for the α-selective glycosylation a more classical Pareto front is observed, consisting of a set of non-dominated solutions.

The advantage of using BO instead of the OVAT approach is that it increases the chance of finding the optimal conditions significantly.^56,57^ However, a disadvantage is that it is more difficult to infer trends from the data, as multiple reaction parameters are being varied at the time, hence making it difficult to pinpoint the effect of changing a specific parameter. Tables 1 and 2 show the experimental conditions and results from optimization campaigns 1 and 2, respectively. Despite multiple parameters being varied across the experiments, it is possible to infer some general trends. For instance, all glycosylation with LiBF_4_ and LiNTf_2_ are β-selective, and all glycosylations with LiI are α-selective, whereas some of the glycosylations with LiPF_6_ are β-selective (Exp. no. 1, 17, 24) and some are α-selective (Exp. no. 11, 56). Interestingly, the presence of molecular sieves seems to be an important factor for the stereoselectivity in some cases. Experiments 1 and 11 are carried out under very similar conditions except for the addition of 3 Å MS to Experiment 1, but a significant difference in selectivity is observed, 31 : 69 for Experiment 1 and 82 : 18 for Experiment 11. However, the α-selectivity cannot be ascribed to the presence of LiPF_6_ and the absence of molecular sieves alone since experiment 24 also is β-selective (28 : 72). Experiment 24 also does not have any additives, but the major solvent is MeCN and the acid catalyst is acetic acid, rather than Et_2_O and oxalic acid as for Experiments 1 and 11. This suggests that some of the variables are interdependent. For the experiments without any acid catalyst (4, 7, 20, 38, 44, 65) the yields are low to moderate, ranging from 3–59%, indicating that lithium-salts can activate the TCA-donor without any additional catalyst, albeit longer reaction times are required for full conversion. This is in accordance with previous studies.^43,58^

**Table 1:** Conditions and results for the experiments carried out during the first optimization campaign optimizing for yield and β-selectivity. Each batch consists of five experiments. Each experiments hypervolume contribution (HV contr.) is given

Exp. no.	Conf.	Li salt	Li salt eq.	Acid	Acceptor eq.	Conc. (M)	Part EtO_2_	Part MeCN	M. S.	Temp (°C)	Yield (%)	Ratio (*β* %)	HV contr. (%)
1	α	LiPF_6_	3.4	Oxalic	1.7	0.18	0.51	0.05	3 Å	25	87	69	60
2	β	LiI	1.5	Acetic	1.3	0.3	0.29	0.06	4 Å	25	96	13	12
3	β	LiI	3.5	TFA	2.8	0.26	0.51	0.08	3 Å	0	69	11	8
4	α	LiI	1.6	None	1.9	0.27	0.11	0.41	3 Å	25	13	36	5
5	α	LiClO_4_	1.7	Acetic	1.4	0.25	0.11	0.81	3 Å	0	64	78	50
6	β	LiNTf_2_	2.2	Oxalic	1.7	0.19	0.41	0.08	3 Å	0	61	63	38
7	β	LiClO_4_	1.7	None	1.8	0.18	0.19	0.11	None	0	59	48	28
8	β	LiClO_4_	3	Acetic	2.2	0.25	0.56	0.16	4 Å	25	74	47	35
9	α	LiBF_4_	2.6	Oxalic	3	0.22	0.75	0.18	4 Å	25	69	73	51
10	α	LiB(C_6_F_5_)_4_	1	Acetic	2.1	0.24	0.65	0.28	5 Å	25	10	0	0
11	α	LiPF_6_	2.8	Oxalic	1.5	0.17	0.33	0.04	None	25	97	18	18
12	β	LiOTf	1.5	Formic	3	0.09	0.44	0.15	None	25	81	49	40
13	β	LiNTf_2_	3.2	Formic	0.8	0.11	0.15	0.6	4 Å	0	28	86	24
14	α	LiOTf	4.6	Oxalic	1.9	0.13	0.05	0.9	4 Å	25	97	64	62
15	β	LiPF_6_	4.1	Acetic	2.2	0.12	0.8	0.12	3 Å	25	98	65	64
16	α	LiNTf_2_	4.8	TFA	1.2	0.12	0.69	0.28	4 Å	0	84	78	66
17	α	LiPF_6_	5	Oxalic	2.5	0.03	0.09	0.87	5 Å	25	93	81	75
18	α	LiClO_4_	5	Formic	1.2	0.28	0.86	0.12	3 Å	25	71	52	37
19	α	LiBF_4_	4.1	Acetic	2.4	0.1	0.98	0.01	5 Å	0	58	59	34
20	α	LiNTf_2_	1.8	None	2.8	0.07	0.31	0.15	3 Å	0	51	86	44
21	α	LiBF_4_	0.5	Formic	1.1	0.18	0.17	0.53	3 Å	25	82	80	66
22	α	LiBF_4_	2.1	Formic	2.3	0.06	0.82	0.04	3 Å	0	62	64	40
23	β	LiOTf	4	TFA	2.5	0.21	0.38	0.49	5 Å	0	76	65	49
24	α	LiPF_6_	4.2	Acetic	2.6	0.15	0.05	0.44	None	0	92	72	66
25	α	LiOTf	3.1	Formic	1.7	0.05	0.13	0.23	5 Å	25	74	61	45
26	β	LiB(C_6_F_5_)_4_	2.5	TFA	1.3	0.08	0.1	0.7	5 Å	25	0	0	0
27	β	LiOTf	3.3	Oxalic	1	0.07	0.52	0.12	4 Å	25	81	49	40
28	β	LiB(C_6_F_5_)_4_	1.5	Formic	2.4	0.16	0.55	0.34	4 Å	0	0	0	0
29	β	LiBF_4_	4.5	Oxalic	1.4	0.26	0.55	0.38	3 Å	25	94	71	67
30	α	LiClO_4_	3.7	Oxalic	2.3	0.23	0.12	0.56	3 Å	25	87	79	69
31	β	LiClO_4_	3.2	Oxalic	2.5	0.13	0.63	0.34	None	25	99	45	45
32	β	LiNTf_2_	4.2	Oxalic	2.7	0.28	0.46	0.25	4 Å	25	13	77	10
33	β	LiNTf_2_	1.2	Oxalic	2.9	0.21	0.04	0.5	4 Å	0	94	80	75
34	α	LiB(C_6_F_5_)_4_	3	TFA	1	0.19	0.03	0.15	4 Å	25	12	0	0
35	β	LiPF_6_	4.1	Formic	2.1	0.05	0.57	0.39	5 Å	25	57	78	45
36	α	LiOTf	3.6	Acetic	2.5	0.17	0.28	0.27	3 Å	0	95	66	62
37	β	LiPF_6_	3.1	Acetic	1.8	0.09	0.4	0.55	4 Å	0	98	77	76
38	β	LiClO_4_	2.5	None	1.3	0.14	0.43	0.49	3 Å	25	3	0	0
39	α	LiBF_4_	2.5	Oxalic	2.2	0.14	0.29	0.63	4 Å	0	73	80	59
40	α	LiB(C_6_F_5_)_4_	1.5	Formic	2.1	0.24	0.87	0.06	4 Å	0	33	69	23
41	α	LiClO_4_	0.8	Acetic	2.5	0.27	0.13	0.71	4 Å	25	58	73	42
42	α	LiBF_4_	2.2	Acetic	1	0.27	0.54	0.29	5 Å	0	68	74	50
43	α	LiOTf	1.8	Acetic	1.9	0.16	0.09	0.47	4 Å	0	30	73	22
44	β	LiB(C_6_F_5_)_4_	1.3	None	2.9	0.24	0.21	0.62	5 Å	0	26	41	11
45	β	LiClO_4_	1.8	Oxalic	1.7	0.31	0.7	0.27	4 Å	25	85	50	42
46	β	LiPF_6_	2.7	Formic	2.5	0.2	0.13	0.48	None	25	99	41	41
47	α	LiBF_4_	0.8	TFA	1.4	0.09	0.38	0.06	3 Å	25	58	74	43
48	α	LiBF_4_	3.5	Acetic	1.4	0.21	0.11	0.73	4 Å	25	85	77	66
49	β	LiClO_4_	5	Formic	1.5	0.18	0.53	0.21	5 Å	25	100	36	36
50	α	LiNTf_2_	3.2	Formic	1	0.22	0.33	0.31	3 Å	25	44	81	36
51	α	LiPF_6_	2.7	Acetic	2.3	0.03	0	0.99	5 Å	0	50	80	40
52	α	LiPF_6_	1.3	Formic	1.2	0.11	0.03	0.11	3 Å	0	63	80	51
53	β	LiClO_4_	0.8	Acetic	2.4	0.1	0.45	0.43	3 Å	0	56	74	41
54	α	LiBF_4_	3.2	Formic	2.8	0.14	0.57	0.29	3 Å	0	78	76	59
55	α	LiBF_4_	1.1	TFA	2.5	0.06	0.02	0.36	4 Å	0	73	80	58

**Table 2:** Conditions and results for the experiments carried out during the second dual optimization campaign. Each batch consists of five experiments. The objectives for the first two experiments in each batch are yield and β-selectivty, whereas the objectives for the remaining three experiments (shaded) are yield and α-selectivty

Exp. no.	Conf.	Li salt	Li salt eq.	Acid	Acceptor eq.	Conc. (M)	Part EtO_2_	Part MeCN	M. S.	Temp (°C)	Yield (%)	Ratio (*β* %)	HV^a^ contr. (%)
56	α	LiPF_6_	4.1	Formic	2.5	0.03	0.01	0.27	None	25	74	33	24 (50)
57	α	LiB(C_6_F_5_)_4_	2.6	Oxalic	1.4	0.1	0.26	0.22	5 Å	25	0	0	0 (0)
58	β	LiClO_4_	2.9	Oxalic	2.3	0.28	0.06	0.06	3 Å	25	99	57	56 (43)
59	β	LiI	4.5	Formic	1.4	0.08	0.31	0.31	4 Å	0	75	15	11 (64)
**60**	**β**	**LiNTf**_**2**_	**4.8**	**Oxalic**	**1.6**	**0.05**	**0.09**	**0.47**	**3 Å**	**25**	**101**	**79**	**80** (21)
61	α	LiNTf_2_	1	TFA	2	0.17	0.22	0.31	4 Å	0	53	84	44 (8)
62	α	LiClO_4_	3.6	TFA	0.9	0.06	0.73	0.2	None	0	53	40	21 (32)
63	α	LiB(C_6_F_5_)_4_	1.6	Oxalic	1.1	0.23	0.48	0.19	4 Å	25	0	0	0 (0)
64	α	LiOTf	3.9	TFA	1.9	0.3	0.24	0.66	3 Å	25	80	66	53 (27)
65	β	LiOTf	4	None	2	0.2	0.44	0.15	4 Å	0	48	46	22 (26)
66	α	LiPF_6_	4.5	Acetic	2.1	0.13	0.58	0.1	4 Å	25	85	73	63 (23)
67	β	LiPF_6_	3	Acetic	2.7	0.18	0.59	0.2	3 Å	0	98	67	65 (32)
**68**	**β**	**LiI**	**1.8**	**Formic**	**1**	**0.18**	**0.3**	**0.12**	**4 Å**	**25**	**76**	**7**	**6** (71)
69	β	LiOTf	4.3	Oxalic	1.1	0.05	0.01	0.59	5 Å	25	12	68	8 (4)
70	α	LiPF_6_	2.2	TFA	1.5	0.11	0.35	0.14	4 Å	25	74	79	58 (16)
**71**	**α**	**LiPF**_**6**_	**3.2**	**Oxalic**	**1.9**	**0.06**	**0.05**	**0.63**	**4 Å**	**25**	**96**	**82**	**78** (17)
72	β	LiBF_4_	4.6	Formic	2.7	0.28	0.33	0.11	3 Å	0	99	60	60 (17)
73	α	LiPF_6_	1.3	Formic	2.4	0.23	0.36	0.63	None	25	80	65	52 (40)
74	α	LiClO_4_	4.1	Oxalic	1.1	0.17	0.24	0.66	5 Å	25	29	64	18 (10)
75	β	LiPF_6_	5	Acetic	1.5	0.3	0.44	0.15	3 Å	0	97	63	62 (36)

a α-Selectivity and yield hypervolume contribution in parenthesis.

### Partial dependence plots analysis

**Fig. 5:**
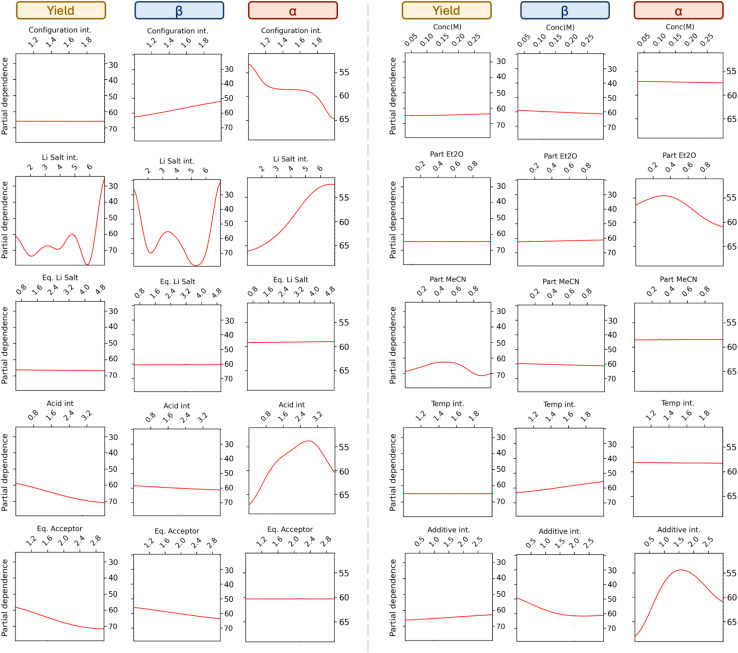
Partial dependence plot for all features and all objectives. The plot shows how each feature influences the yield, percentage β-anomer, or percentage α-anomer, while averaging out the effects of all other features. Note that the objective function is approximated as a continuous function even in the case of discrete parameters.

To get a more systematic understanding of the influence of each parameter we turned to partial dependence plots, which is a way of visualizing the relationship between selected parameters and the predicted outcome, as the plots show the effect of each parameter on each objective when averaging out all other parameters.^59,60^ The estimated effect of each parameter on yield, β-selectivity, and α-selectivity are shown in Fig. 5. It should be noted that the partial dependence of the discrete parameters is illustrated as a continuous function, thus some parts of these graphs do not carry physical meaning. Starting from the top left, it is seen that the anomeric configuration of the glycosyl donor does not influence the yield. However, an inversely correlated effect is seen on the stereoselectivities, indicating that some of the reactions might be stereospecific. For the lithium salts, the identity of the salt influences both the yield and the selectivity. The β-selectivity plot shows the highest β-selectivities for salt 2, 5, and 6, which are LiBF_4_, LiNTf_2_, and LiPF_6_, respectively. The α-selectivity plot shows a maximum at lithium salt 1 (LiI). These trends are in line with the observations from the raw data discussed earlier. Interestingly, a close to linear response between the lithium salt PCA integer assignment and the α-selectivity is observed, indicating that the descriptors used for the PCA are a good measure for α-selectivity.

The amount of lithium salt does not seem to have an influence on any of the objectives. The acid plots suggest that the stronger the acid, the higher the yield, which might be due to faster reaction times and the absence of rebound product between the glycosyl donor and conjugate base of the acid.^61^ The acid also seems to have an impact on the α-selectivity but with no clear trend, while the influence on the β-selectivity is minor. A higher amount of acceptor results in a higher yield, which might also be related to faster reaction times. On the top right, the influence of the concentration is shown, which only seems to have a minor impact on all the objectives. The amount of ether solvent improves the α-selectivity in agreement with known solvent effects. However, interestingly the effect of increasing the amount of acetonitrile in the solvent only shows a very minor increase in β-selectivity, though, this is in agreement with previous observations showing that the presence of other additives diminishes the acetonitrile effect.^62^ The partial dependence plot indicates a slight increase in β-selectivity is observed at lower temperatures.

Lastly, the effect of additives is shown. Noticeably, having no additives (additives integer equal to 0) increases the α-selectivity, which is also supported by the earlier comparison of Experiment 1 and 11 (Table 1). To fully understand the effect of the parameters, the reaction mechanism(s) and evolvement of all reaction components would have to be elucidated.

**Fig. 6:**
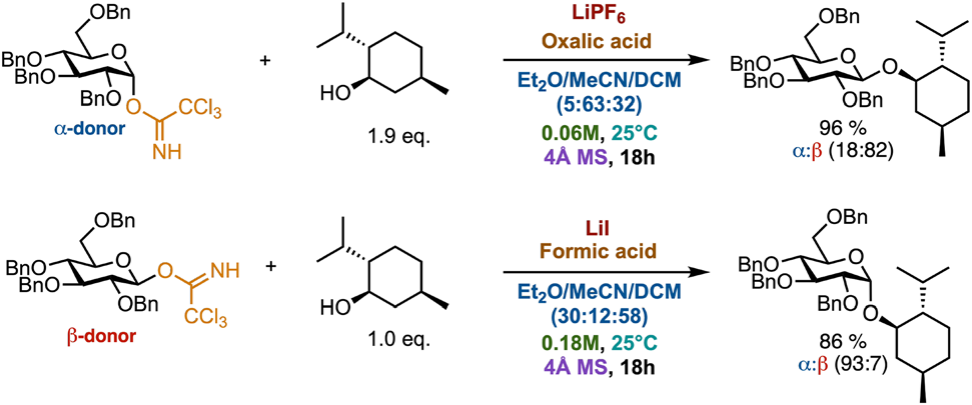
Picked lead reactions for stereoselective lithium salt directed glycosylations. The top reaction depicts Experiment 71 in Table 2 and the bottom reaction depicts Experiment 68 in Table 2.

Based on the results we propose the conditions from Experiment 71 in Table 1 as lead for new glycosylation methods for β-selective lithium salt directed glycosylation, and the conditions from Experiment 68 (Table 1) as a new glycosylation method for α-selective lithium salt directed glycosylation. The lead reactions are depicted in Fig. 6.

From previous studies, it seems plausible that a glycosyl iodide is formed as an intermediate in the LiI-directed glycosylations, which leads to α-selectivity through Curtin–Hammett kinetics.^63^ Similar intermediates and stereoselectivity have been observed for NIS/TfOH-activated glycosylations with thioglycosides.^64^ The high β-selectivity observed for 60 and 71 also aligns with the formation of either a covalent adduct or a contact ion pair between the counterion and the putative glycosyl cation. The highly electronegative counterions would favor the axial position due to the anomeric effect leading to attack by the nucleophile on the equatorial position.^50^ However, all the counterions are highly electronegative, thus the exact role of the lithium salts, acids, and molecular sieves remains to be elucidated.

### Assessing novelty

There is no ubiquitous way of establishing the novelty of a reaction, and the terms new reaction and novel reaction are used ambiguously.^5,65–68^ The demand for a reaction to be novel ranges from only one component being new to unprecedented reactivity.^5,65–68^ To assess the novelty of our discovery we turned to a definition by Cronin and co-workers,^65^ who state, that for a discovery to be novel it has to be repeatable, not observed previously, and non-predictable. We argue that the discovered lithium salt-directed glycosylations fulfill all these demands, as the changes in stereoselectivity based on lithium salts, molecular sieves, *etc.* are non-obvious and unpredictable. However, even with this definition, the term novel reaction is still not entirely unambiguous. The reactions described in this study fall under the known category glycosylation reactions, which in terms of reactivity can by itself not be described as a “novel reaction”, as glycosylation reactions are mostly S_N_1 or S_N_2-reactions *i.e.* the reactivity is well-known. We therefore chose to evaluate if our discovery is a novel reaction based on the position in reaction space.

**Fig. 7:**
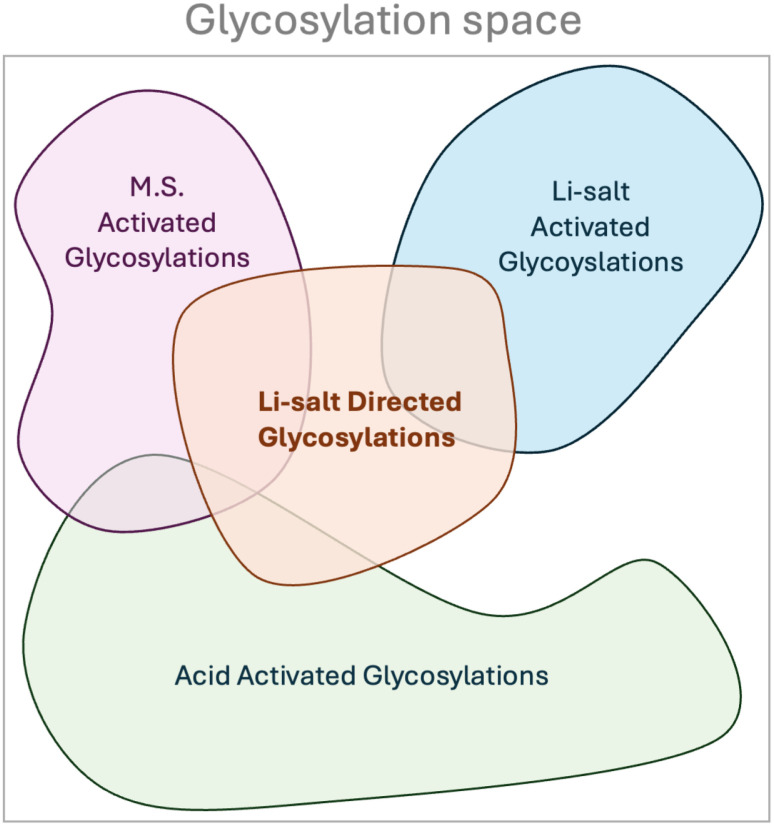
Depiction of glycosylation space which is a subspace of reaction space. It is illustrated that since both lithium salt, molecular sieves, and acid are important for the outcome of lithium salt-directed glycosylation, these comprise a previously undiscovered part of the glycosylation space.

It is clear from the partial dependence plot that both the lithium salt, acid, and additive are important for the outcome of the reaction. Thus the methodologies cannot be classed into well-known procedures like acid-activated glycosylations,^49,69^ acid-washed molecular sieves activated glycosylation,^70^ or lithium salt activated.^43,58,62,71^ Instead, the methodology of lithium salt-directed glycosylation encapsulates a previously unknown part of the glycosylation reaction space as illustrated in Fig. 7. To the best of our knowledge, this is the first example of Bayesian optimization being used for this degree of reaction discovery.

## Conclusion

We demonstrate a new workflow for identifying lead reactions in method development within a broad reaction class. This is done utilizing Bayesian optimization as a tool for discovering novel stereoselective glycosylation methodologies. Specifically, we find that a combination of lithium salt and mild acid promotes the reaction of a glycosyl TCA with l-menthol, resulting in high yields. The anomeric selectivity can be directed by the choice of lithium salt and the additional reaction conditions. We also show how partial dependence plots can be used to visualize the influence of each reaction parameter on the yield and stereoselectivity. From the plots, we can infer trends and gain mechanistic insights, in a similar manner to how OVAT data is analyzed.

## Author contributions

NVF designed and performed the experiments and wrote the draft manuscript. CMP securred funding, supervised and contributed to the discussion of the results and to the revision of the manuscript.

## Conflicts of interest

The authors declare no competing interests.

## Supplementary Material

SC-016-D5SC03244J-s001

## Data Availability

Natasha Videcrantz Faurschou 2024 GitHub “GlycoTools” https://github.com/NatashaVF/GlycoTools.
