# Supplementary material for: Discovery of novel glycosylation methods using Bayesian optimization: lithium salt directed stereoselective glycosylations
Source: Chem Sci. 2025 Jul 8;16(33):15056–65. doi: 10.1039/d5sc03244j (PMC12278502; doi:10.1039/d5sc03244j)
Supplement: SC-016-D5SC03244J-s001 [file SC-016-D5SC03244J-s001.pdf]

Supplementary Information (SI) for Chemical Science.
This journal is © The Royal Society of Chemistry 2025

Reaction Discovery Using Bayesian Optimization: Lithium Salt Directed Stereoselective Glycosylations

Natasha Videcrantz Faurschou† and Christian Marcus Pedersen†*

May 2, 2025

Contents

1 Compound Characterization List **2**

2 Experimentals **2**

2.1 General . . . 2

2.2 Synthesis and Characterization . . . 2

2.3 Optimization Campaigns . . . 5

3 PCA of lithium salts **5**

4 Dimensionality Reduction **7**

5 NMR spectra **8**

6 References **14**

1 Compound Characterization List

No.	Structure	New	^{1}H NMR	^{13}C NMR	COSY	HSQC	HRMS	R_f
**1**	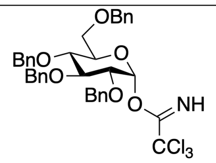	−	+	+	−	−	+	+
**2**	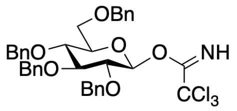	−	+	+	+	−	+	+
**3**	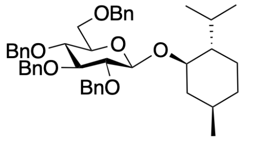	−	+	+	+	+	+	+
**4**	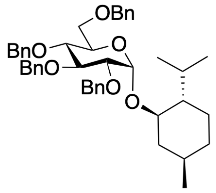	−	+	+	+	+	+	+

Note: + equals true or included, and − equals false or not included.

2 Experimentals

2.1 General

All chemicals have been used as purchased from the supplier without further purification. Reactions have been carried out with dry solvents, either collected from an Innovative Technology PS-MD-05 solvent drying system or by drying over 4 Å molecular sieves.

Reactions were monitored by TLC using aluminum sheets coated with silica (Merck F_{254}-plates). The spots were visualized using UV and staining with 10% H_2SO_4 in ethanol. All reactions were carried out in flame-dried glassware. Flash column chromatography was performed with silica gel (40-63 μm).

HRMS values were obtained using MALDI and the mass spectra were recorded using a Bruker SolariX XR 7T ESI/MALDI-FT-ICR-MS instrument. ^{1}H-NMR and ^{13}C-NMR spectra were recorded at 500 MHz and 125 MHz, respectively, using a Bruker 500 MHz Ultra Shield Plus instrument with a cryoprobe.

2.2 Synthesis and Characterization

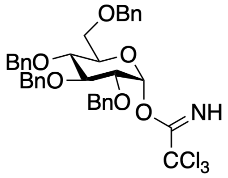

1

2,3,4,6-Tetra-*O*-benzyl-α-D-glucopyranosyl trichloroacetimidate

1,8-diazabicyclo(5.4.0)undec-7-ene (0.05 mL, 335 nmol) was added to a solution of 2,3,4,6-tetra-*O*-

benzyl-α/β-D-glucopyranose (2.50g, 4.62 mmol) and trichloroacetonitrile (5.0 mL, 46 mmol) in dry dichloromethane (20 mL). After two hours TLC indicated full conversion and the reaction was stopped. The reaction mixture was concentrated and purified by flash column chromatography with a mixture of EtOAc/heptane as the eluent to yield 0.87 g of compound 1 (28%).

^{1}**H NMR** (500 MHz, $CDCl_3$) δ: 8.58 (s, 1H, NH), 7.38 - 7.22 (m, 18H, Ar), 7.18 - 7.11 (m, 2H, Ar), 6.53 (d, J = 3.4 Hz, 1H, H-1), 4.96 (d, J = 11.0 Hz, 1H, CH_2(Bn)), 4.85 (d, J = 10.6 Hz, 1H, CH_2(Bn)), 4.83 (d, J = 11.0 Hz, 1H, CH_2(Bn)), 4.75 (d, J = 11.8 Hz, 1H, CH_2(Bn)), 4.68 (d, J = 11.7 Hz, 1H, CH_2(Bn)), 4.60 (d, J = 12.0 Hz, 1H, CH_2(Bn)), 4.53 (d, J = 10.6 Hz, 1H, CH_2(Bn)), 4.47 (d, J = 12.1 Hz, 1H, CH_2(Bn)), 4.05 (t, J = 9.4 Hz, 1H, H-3), 3.99 (ddd, J = 10.1, 3.3, 1.9 Hz, 1H, H-5), 3.83 - 3.74 (m, 3H, H-2,H-4,H-6a), 3.67 (dd, J = 10.9, 2.0 Hz, 1H, H-6b).

^{13}**C NMR** (126 MHz, $CDCl_3$) δ: 161.5, 138.8, 138.2, 138.1, 138.0, 128.6, 128.5, 128.5, 128.2, 128.2, 128.1, 128.1, 128.0, 127.9, 127.9, 127.8, 127.8, 94.5, 91.4, 81.5, 79.5, 75.8, 75.5, 73.6, 73.3, 73.0, 68.2.

1 aliphatic signal is not accounted for in ^{13}*C NMR, likely due to overlapping signals.*

The NMR data are in accordance with literature.[1]

$\mathbf{R}_f$: 3:7 (EtOAc/heptane) = 0.59

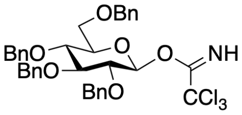

2

2,3,4,6-Tetra-*O*-benzyl-β-D-glucopyranosyl trichloroacetimidate
K_2CO_3 (1.98 g, 14.29 mmol) was added to a stirred solution of 2,3,4,6-tetra-*O*-benzyl-α/β-D-glucopyranose (2.00 g, 3.70 mmol) and trichloroacetonitrile (1.5 mL, 15 mmol) in dry dichloromethane (18 mL). After stirring for one hour the reaction was stopped. The reaction mixture was filtered, concentrated, and purified by flash column chromatography (1:9 → 1:6 EtOAc/heptane, packed with 1.5% Et_3N) to yield **1** in a 1:16 α/β ratio as a clear syrup (0.825 g, 1.20 mmol, 33 %).

^{1}**H NMR** (500 MHz, $CDCl_3$) δ: 8.73 (s, 1H, NH), 7.40 - 7.27 (m, 18H, Ar), 7.23 - 7.15 (m, 2H, Ar), 5.87 - 5.80 (m, 1H, H-1), 4.97 (d, J = 10.9 Hz, 1H, CH_2(Bn)), 4.94 (d, J = 11.1 Hz, 1H, CH_2(Bn)), 4.87 - 4.82 (m, 2H, 2xCH_2(Bn)), 4.79 (d, J = 10.9 Hz, 1H, CH_2(Bn)), 4.68 - 4.53 (m, 3H, 3xCH_2(Bn)), 3.86 - 3.72 (m, 5H, H-2/H-3/H-5/H-6a/H-6b), 3.70 - 3.61 (m, 1H, H-2/H-3/H-5/H-6a/H-6b)

^{13}**C NMR** (126 MHz, $CDCl_3$) δ: 161.2, 138.5, 138.1, 138.0, 138.0, 128.5, 128.4, 128.4, 128.4, 128.1, 128.0, 128.0, 127.9, 127.9, 127.8, 127.7, 127.7, 98.4, 91.0, 84.6, 81.0, 75.9, 75.6, 75.0, 74.9, 73.4, 68.2.

The NMR data are in accordance with literature.[1]

$\mathbf{R}_f$: 1:1 (EtOAc/heptane) = 0.48

HRMS (MALDI) m/z calcd for $C_{36}H_{36}Cl_3NO_6Na+$ 706.15004; Found 706.14927

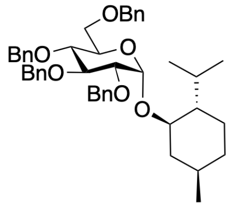

3

L-Menthyl 2,3,4,6-tetra-*O*-benzyl-α-D-glucopyranoside

^{1}**H NMR** (500 MHz, CDCl$_3$) δ: 7.33 - 7.15 (m, 18H, Ar), 7.09 - 7.01 (m, 2H, Ar), 4.95 (d, J = 3.7 Hz, 1H, H-1), 4.91 (d, J = 10.9 Hz, 1H, CH$_2$(Bn)), 4.79 - 4.72 (m, 2H, CH$_2$(Bn)), 4.68 - 4.55 (m, 3H, CH$_2$(Bn)), 4.44 - 4.33 (m, 2H, CH$_2$(Bn)), 3.94 (t, J = 9.3 Hz, 1H, H-3), 3.90 (ddd, J = 10.3, 3.7, 2.2 Hz, 1H, H-5), 3.68 (dd, J = 10.4, 3.7 Hz, 1H, H-6a), 3.61 - 3.54 (m, 2H, H-4, H-6b), 3.48 (dd, J = 9.7, 3.6 Hz, 1H, H-2), 3.28 (td, J = 10.6, 4.4 Hz, 1H, OCH), 2.34 (m, 1H, CH), 2.04 (m, 1H, CH$_2$), 1.59 - 1.48 (m, 2H, 2xCH$_2$), 1.34 - 1.15 (m, 2H, 2xCH), 0.96 (q, J = 11.8 Hz, 1H, CH$_2$), 0.92 - 0.80 (m, 8H, CH), 0.81 - 0.67 (m, 7H, CH, 2xCH$_3$), 0.63 (d, J = 6.9 Hz, 3H, CH$_3$).

^{13}**C NMR** (126 MHz, CDCl$_3$) δ: 139.0, 138.5, 138.4, 138.1, 128.5, 128.5, 128.5, 128.5, 128.4, 128.1, 127.8, 127.8, 127.7, 98.7 (C-1), 82.1, 81.0, 80.6, 78.1, 75.7, 75.2, 73.6, 73.3, 70.3, 68.6, 48.9, 43.2, 34.3, 31.9, 24.7, 23.0, 22.4, 21.3, 16.2.
3 aromatic signals are not accounted for, likely due to overlapping signals.

The NMR data are in accordance with literature.[2]

R$_f$: 3:7 (EtOAc/heptane) = 0.62

HRMS (MALDI) m/z calcd for $C_{44}H_{54}O_6Na+$ 701.38126; Found 701.38102

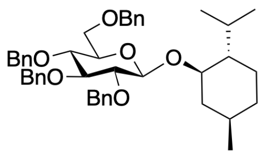

4

L-Menthyl 2,3,4,6-tetra-*O*-benzyl-β-D-glucopyranoside

^{1}**H NMR** (500 MHz, CDCl$_3$) δ: 7.30 - 7.17 (m, 18H, Ar), 7.16 - 7.09 (m, 2H, Ar), 4.91 - 4.82 (m, 2H, 2xCH$_2$(Bn)), 4.76 - 4.70 (m, 2H, 2xCH$_2$(Bn)), 4.61 (d, J = 10.8 Hz, 1H, CH$_2$(Bn)), 4.56 - 4.44 (m, 2H, 3xCH$_2$(Bn)), 4.40 (d, J = 7.8 Hz, 1H, H-1), 3.62 (d, J = 3.3 Hz, 2H, H-6a, H-6b), 3.59 - 3.49 (m, 2H, H-3, H-4), 3.42 (td, J = 10.7, 4.1 Hz, 1H, OCH), 3.38 - 3.31 (m, 2H, H-2, H-5), 2.28 (m, 1H, CH), 2.06 (m, 1H, CH$_2$), 1.66 - 1.55 (m, 2H, 2xCH$_2$), 1.34 - 1.24 (m, 1H, CH), 1.19 (m, 1H, CH), 0.99 - 0.79 (m, 9H, 3xCH$_2$, 2xCH$_3$), 0.75 (d, J = 6.9 Hz, 3H, CH$_3$).

^{13}**C NMR** (126 MHz, CDCl$_3$) δ: 138.9, 138.6, 138.4, 138.2, 128.6, 128.5, 128.5, 128.5, 128.3, 128.0, 127.9, 127.8 127.8, 127.7, 101.0 (C-1), 85.1, 82.3, 78.0, 78.0, 75.8, 75.2, 75.0, 74.9, 73.8, 69.4, 48.2, 41.1, 34.5, 31.6, 25.3, 23.2, 22.4, 21.3, 16.0.
2 aromatic signals are not accounted for, likely due to overlapping signals.

The NMR data are in accordance with literature.[2]

R$_f$: 3:7 (EtOAc/heptane) = 0.71

HRMS (MALDI) m/z calcd for $C_{44}H_{54}O_6Na+$ 701.38126; Found 701.38102x

2.3 Optimization Campaigns

50 mg of TCA was added to a vial which had been dried in an oven and dry solvent(s) were added. The mixture was stirred, and L-menthol was added, followed by the addition of 0.5 mL molecular sieves which were flame dried under vacuum and stored at 200 degrees C. Lithium salt was added to the mixture and it was stirred at either 0°C or 25°C, and 1 drop ($\sim$ 4 μL) of acid catalyst was added. The reaction mixture was then stirred at 0°C or 25°C for 18 hours, whereafter the reaction mixture was diluted with ethyl acetate, filtrated, washed twice with 1 M NaOH, and once with brine. The organic phase was dried over Na_2SO_4 and concentrated in vacuo. 800 of a 0.0238 M solution of 1,4-dimethoxybenzene in $CDCl_3$ was added to the concentrate and both ^{1}H-NMR and ^{13}C-NMR were recorded. In the experiments with $LiNTf_2$ as the lithium salt, precipitate was observed upon addition of the $CDCl_3$ solution, and the solution was therefore filtrated before recording the NMR-spectra.

3 PCA of lithium salts

The principal component analysis (PCA) was carried out using the descriptors shown in 1. The source for molecular weight and complexity is Pubchem. Melting points are from https://www.chembk.com/ and pK_aH of the anions conjugate acid from *J. Org. Chem.* **2011**, *76*, 2, 391 - 395. The pK_a of HPF_6 and $HB(C_6F_5)_4$ are estimated to be -18 as these superacids are too strong for a pKaH in DCE to be measured.[3,4] All features were standardized and the PCA was carried out using the following hyperparameters: *'copy': True, 'iterated_power': 'auto', 'n_components': 2, 'n_oversamples': 10, 'power_iteration_normalizer': 'auto', 'random_state': None, 'svd_solver': 'auto', 'tol': 0.0, 'whiten': False.*

Compound	MW (g/mol)	pK_{aH}(DCE)	Melting point (°C)	Complexity
$LiClO_4$	106.4	-13	236	118
$LiNTf_2$	287.1	-11.9	236	379
$LiPF_6$	151.9	-18	200	67.1
$LiBF_4$	93.8	-10.3	296.5	23
$LiB(C_6F_5)_4$ (Etherate)	685.98	-18	120	815
LiI	133.9	-7.7	446	2
LiOTf	156	-11.4	423	163

Table 1: Descriptors and their values used for the principle component analysis.

Figure 1 shows the resulting PCA, and integers were assigned according to the first principal component.

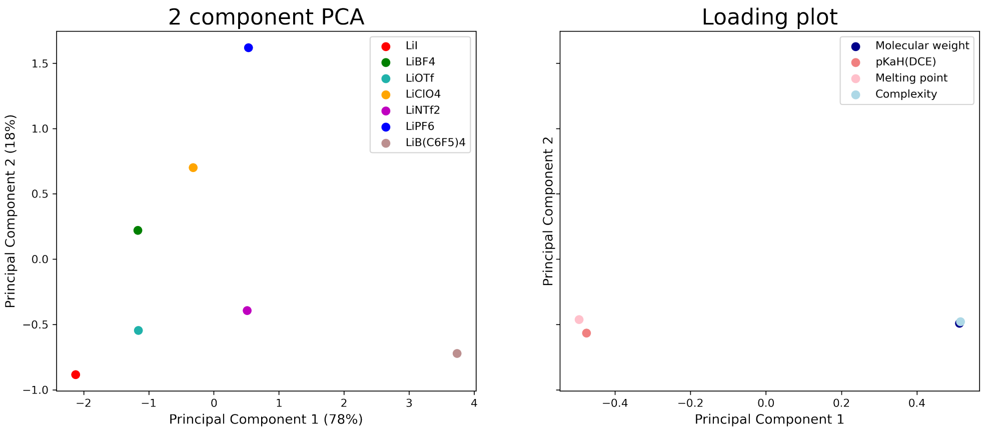

Figure 1: Left: PCA plot, showing the first two principal components and the percentage variance described by each. Right: Loading plot showing the influence of each of the descriptors on the two first principal components.

4 Dimensionality Reduction

The reaction conditions were attempted clustered using dimensionality reduction, however, no clear trends were observed as seen below.

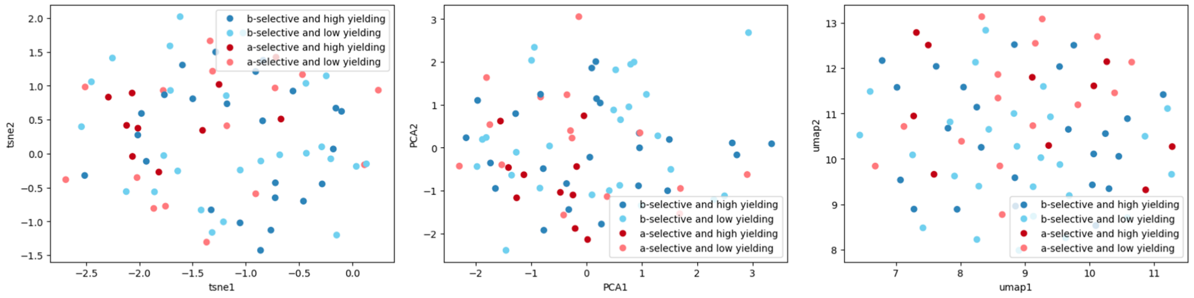

Figure 2: Three types of dimensionality reduction (PCA, TSNE, UMAP) of the reaction conditions for all experiments.

5 NMR spectra

2,3,4,6-Tetra-*O*-benzyl-α-D-glycopyranosyl trichloroacetimidate (1)

^{1}H NMR (500MHz, $CDCl_3$)

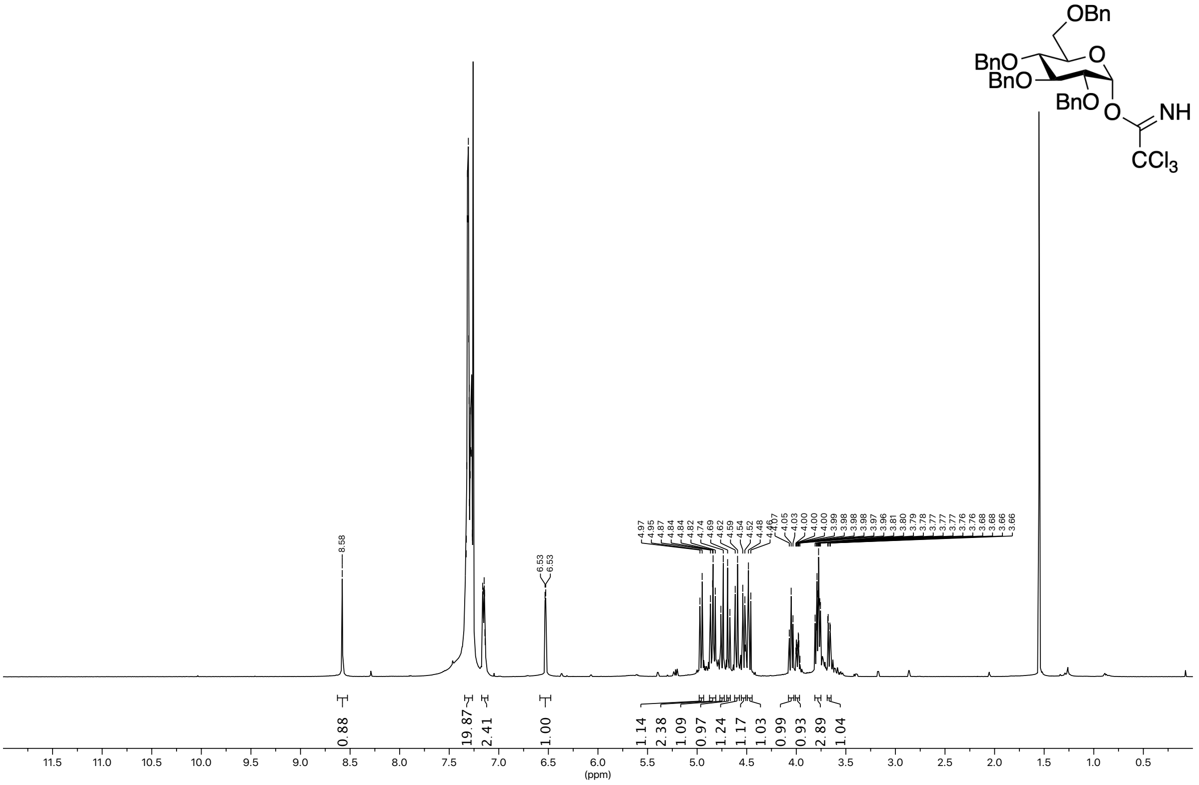

^{13}C NMR (126MHz, $CDCl_3$)

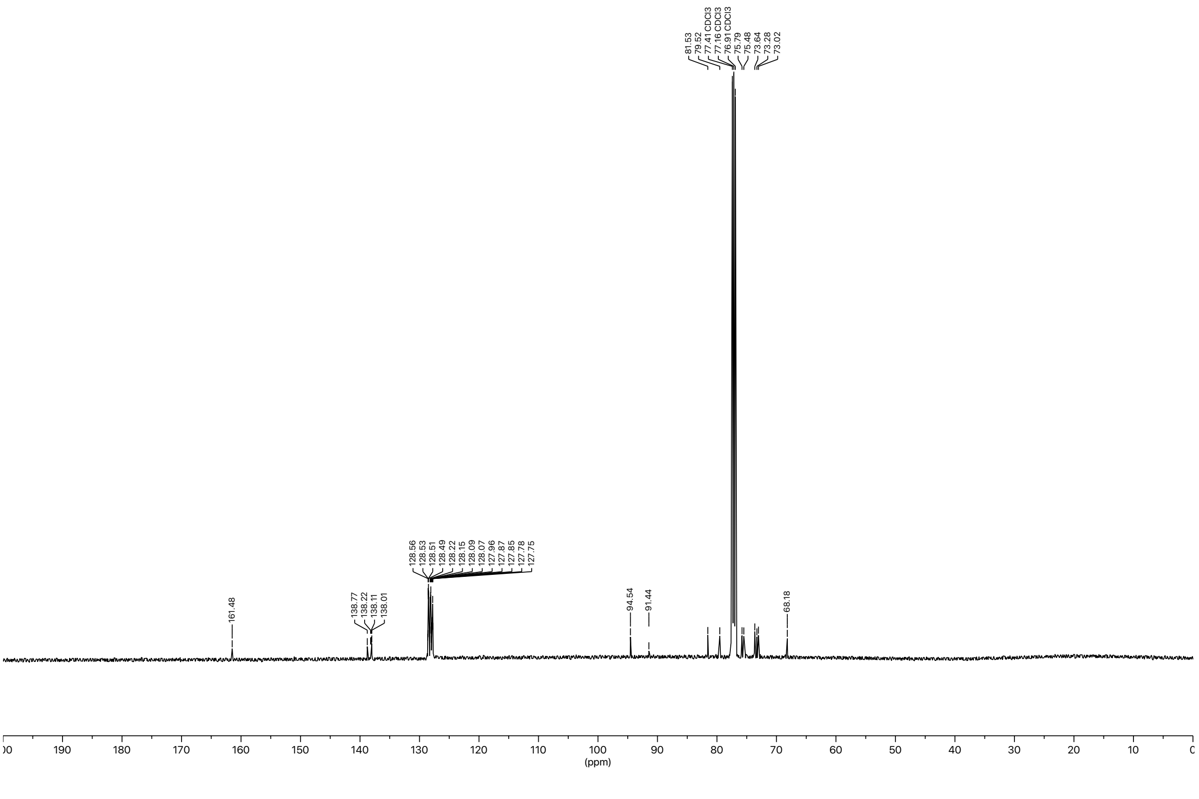

COSY ^{1}H NMR (500MHz, $CDCl_3$)

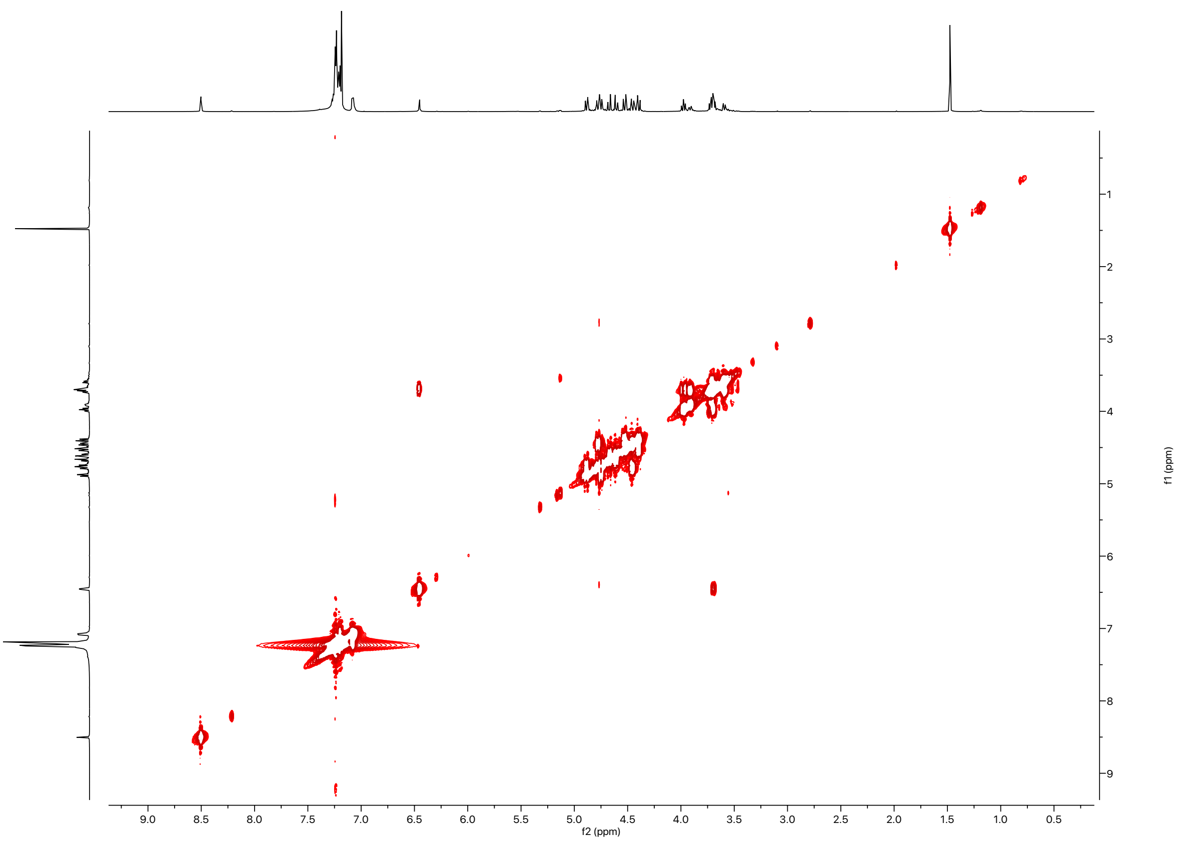

2,3,4,6-Tetra-*O*-benzyl-β-D-glycopyranosyl trichloroacetimidate (2)

^{1}H NMR (500MHz, $CDCl_3$)

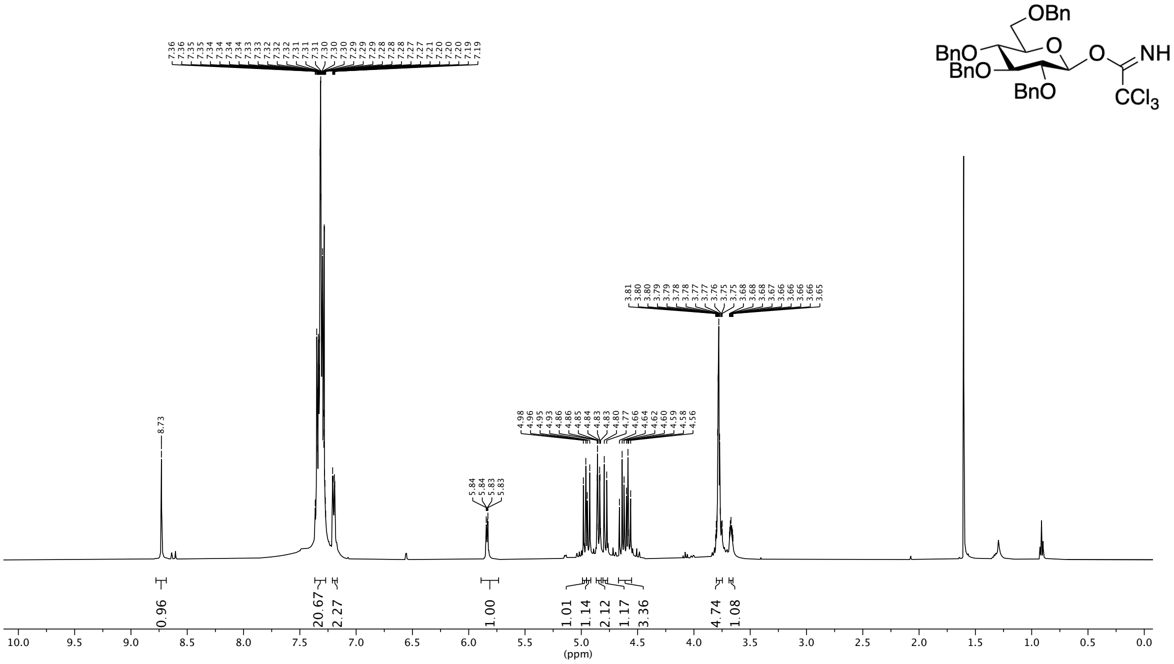

^{13}C NMR (126MHz, $CDCl_3$)

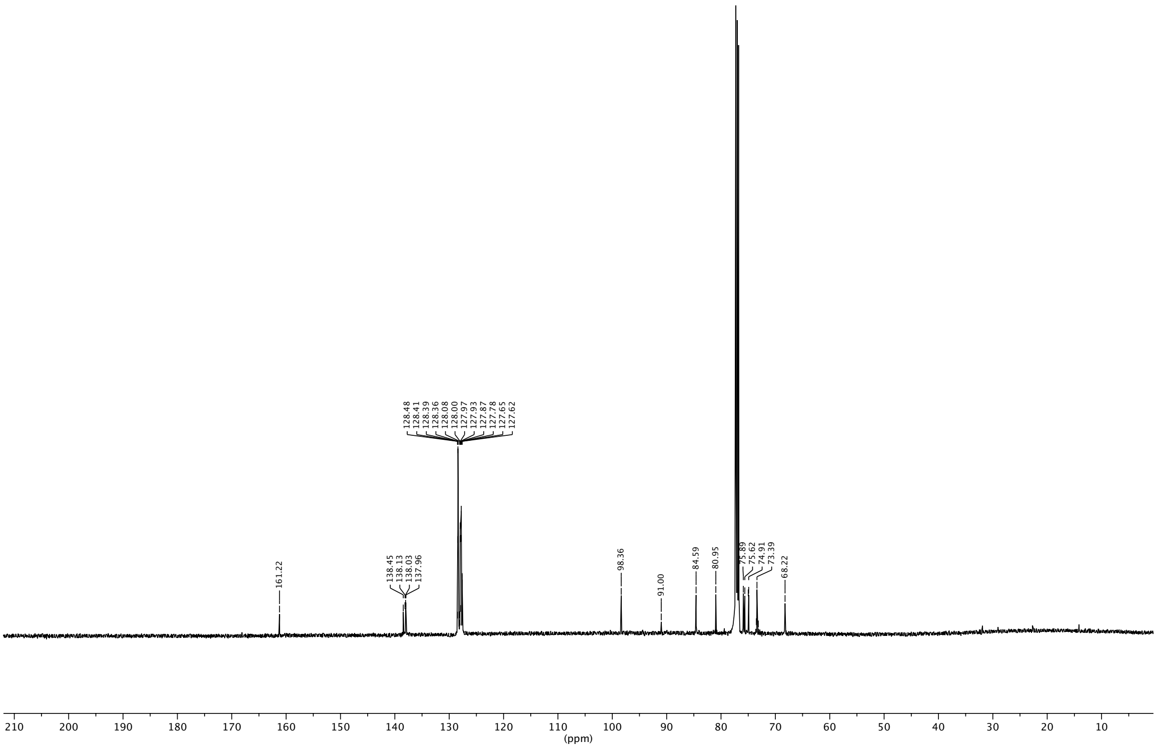

L-Menthyl 2,3,4,6-tetra-*O*-benzyl-α-D-glycopyranoside (3)
^{1}H NMR (500MHz, $CDCl_3$)

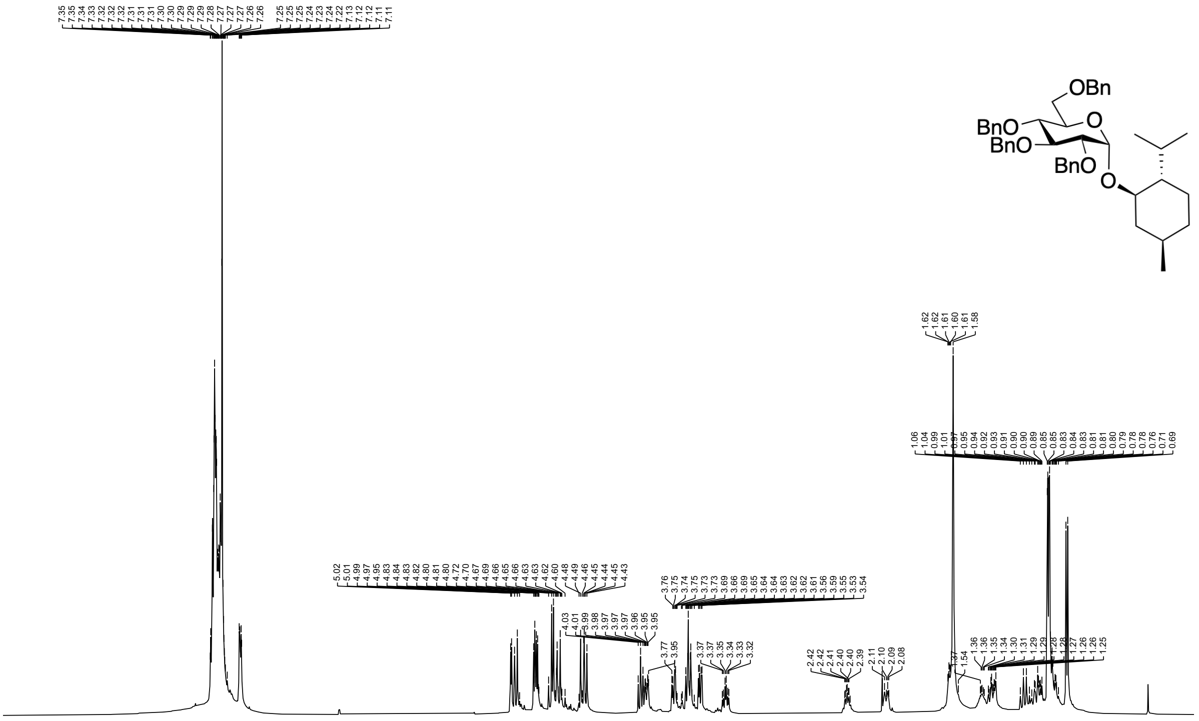

^{13}C NMR (126MHz, $CDCl_3$)

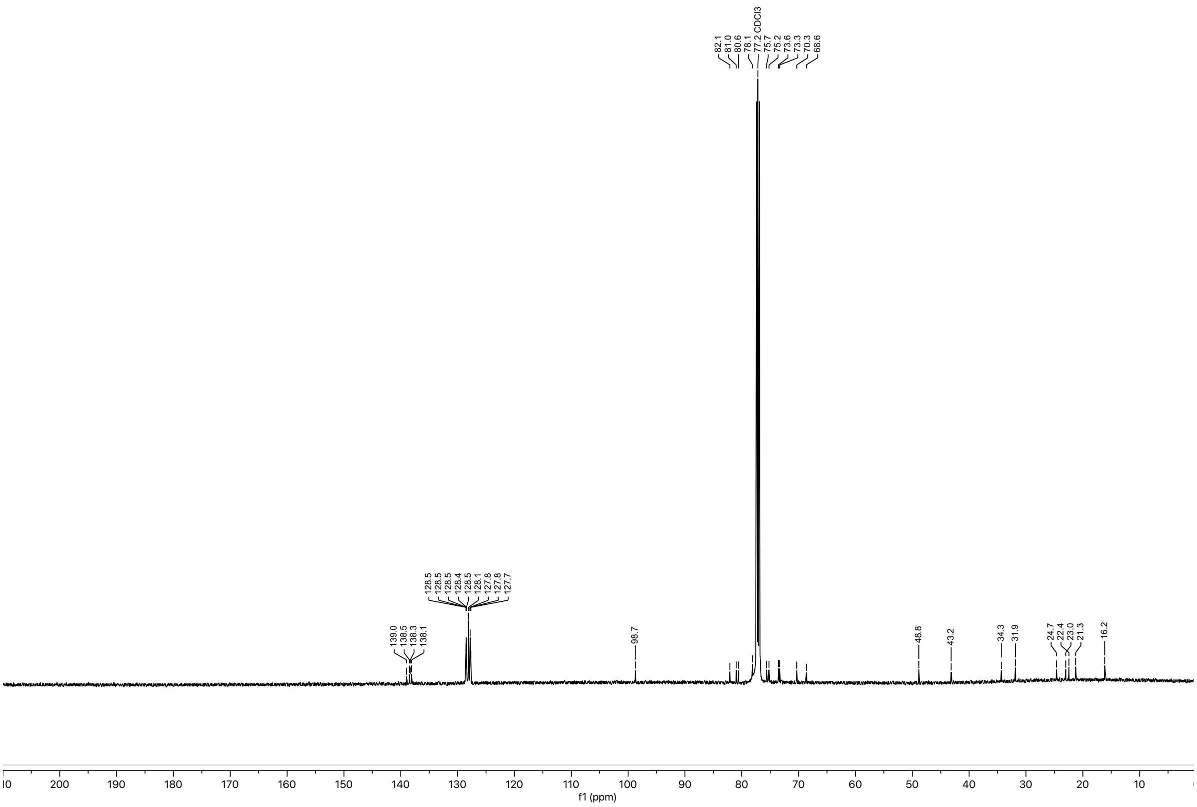

COSY H^1 NMR (500MHz, $CDCl_3$)

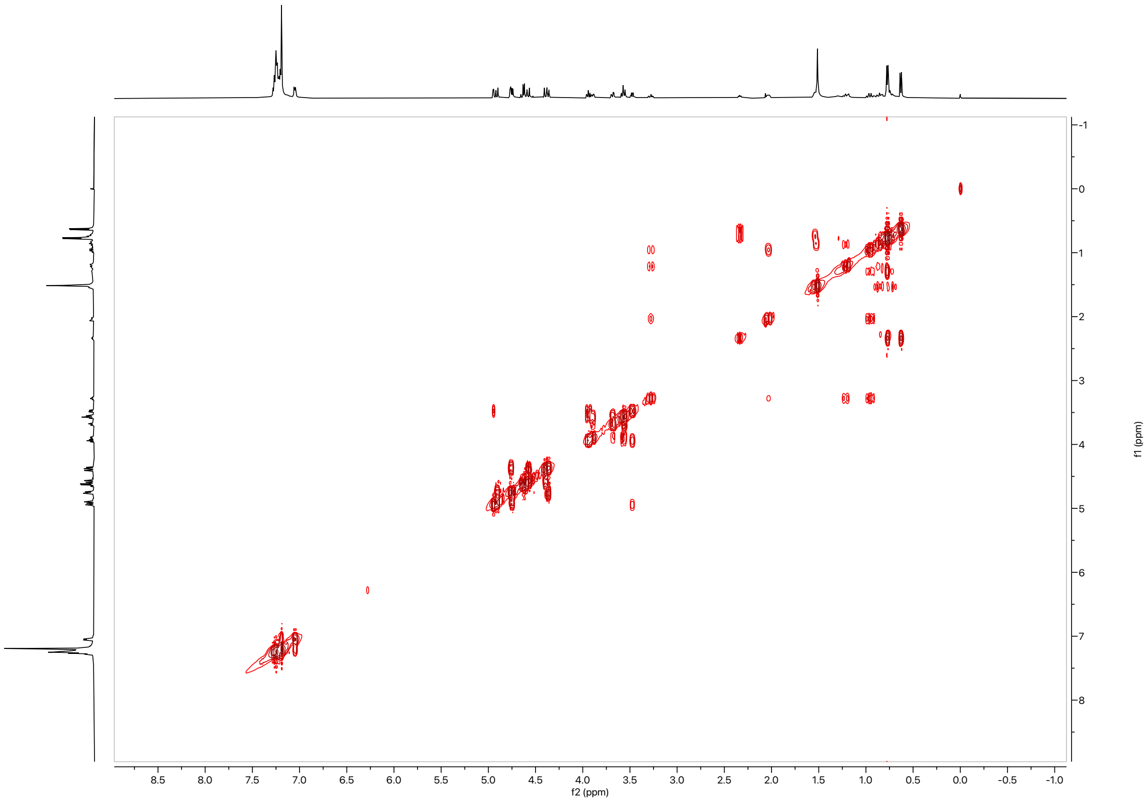

HSQC H[1] NMR (500MHz, $CDCl_3$), ^{13}C NMR (126MHz, $CDCl_3$)

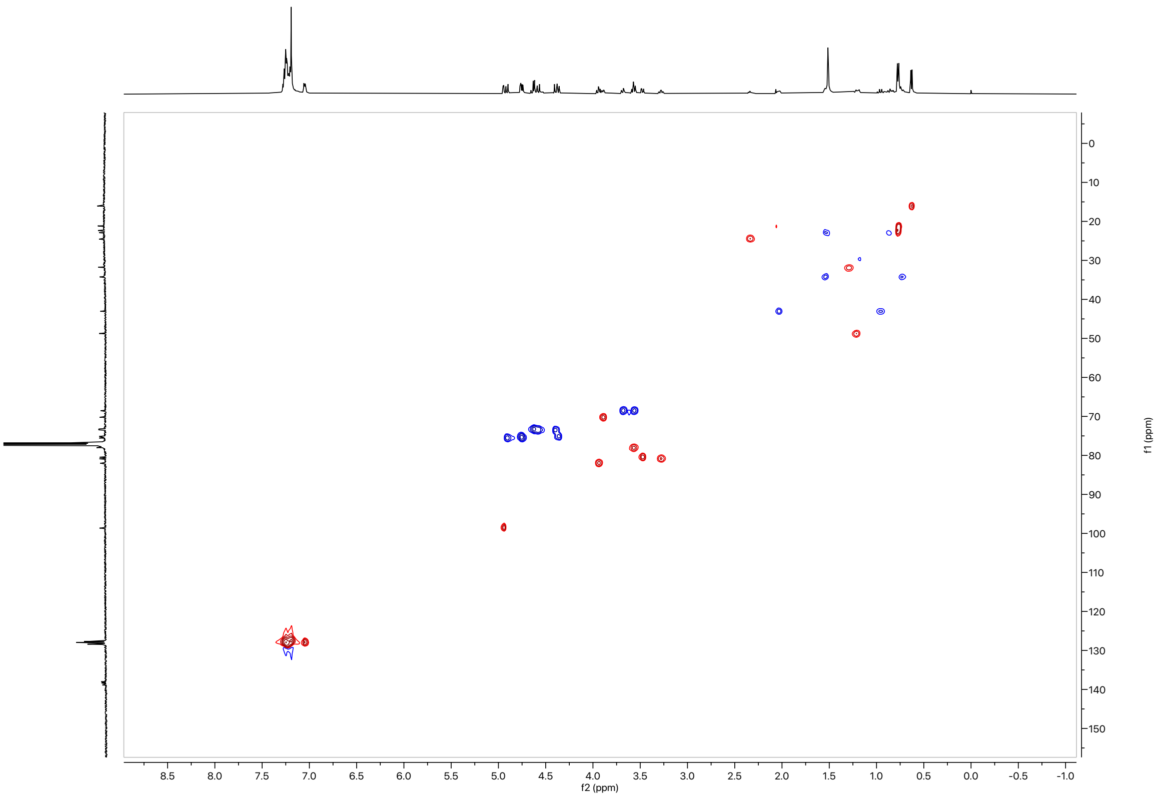

L-Menthyl 2,3,4,6-tetra-*O*-benzyl-β-D-glycopyranoside (4)

^1H NMR (500MHz, $CDCl_3$)

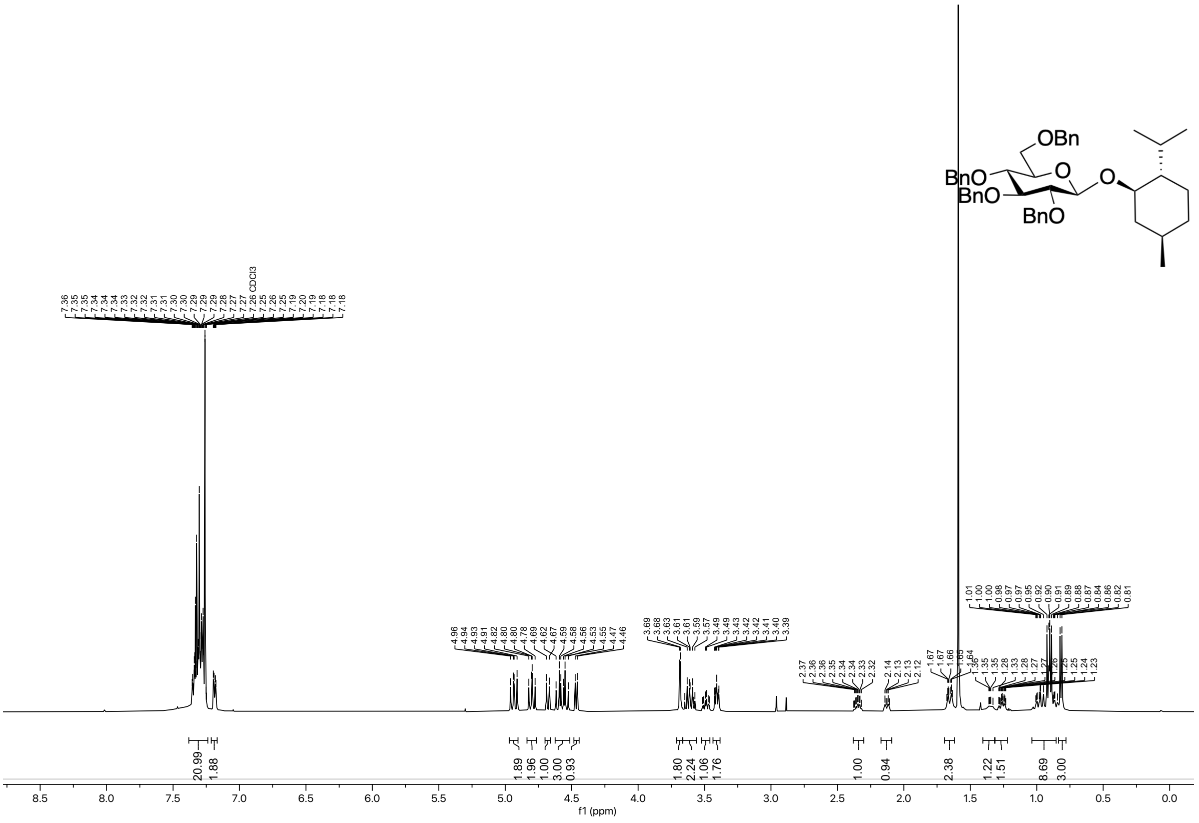

^{13}C NMR (126MHz, $CDCl_3$)

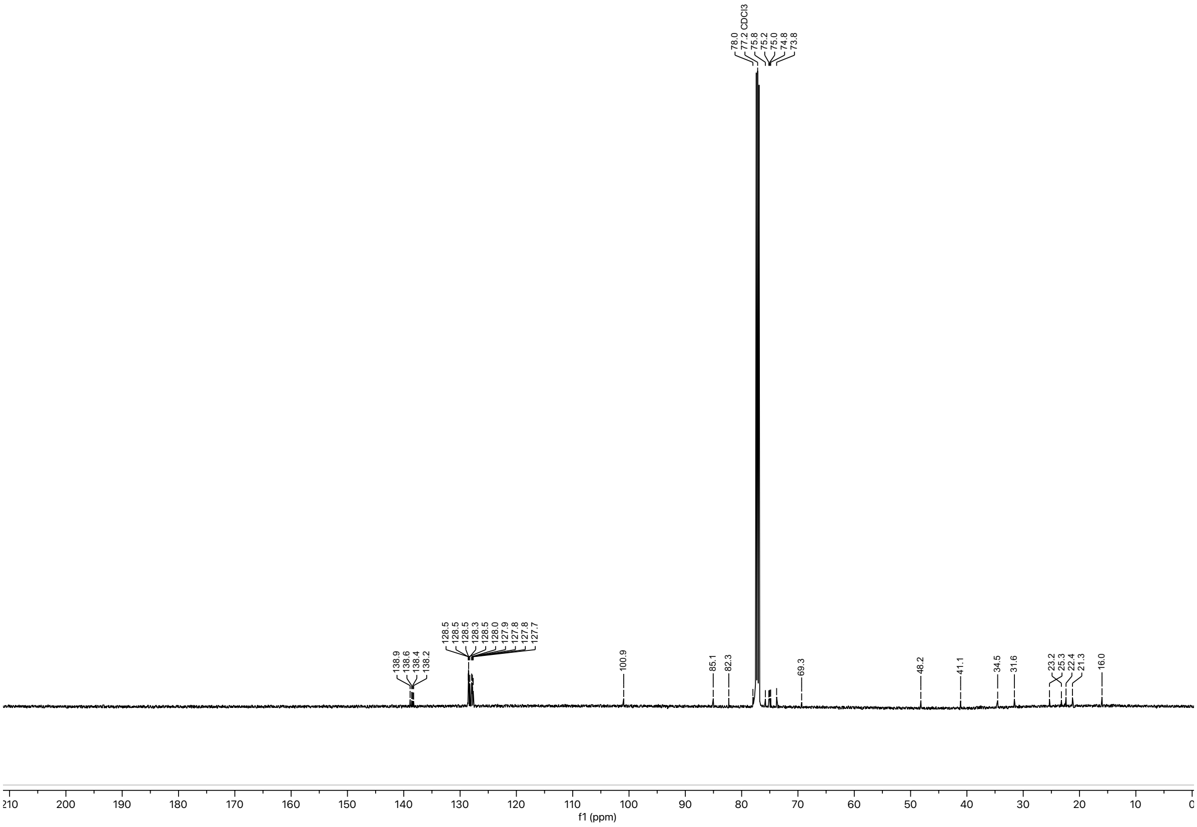

COSY ^1H NMR (500MHz, $CDCl_3$)

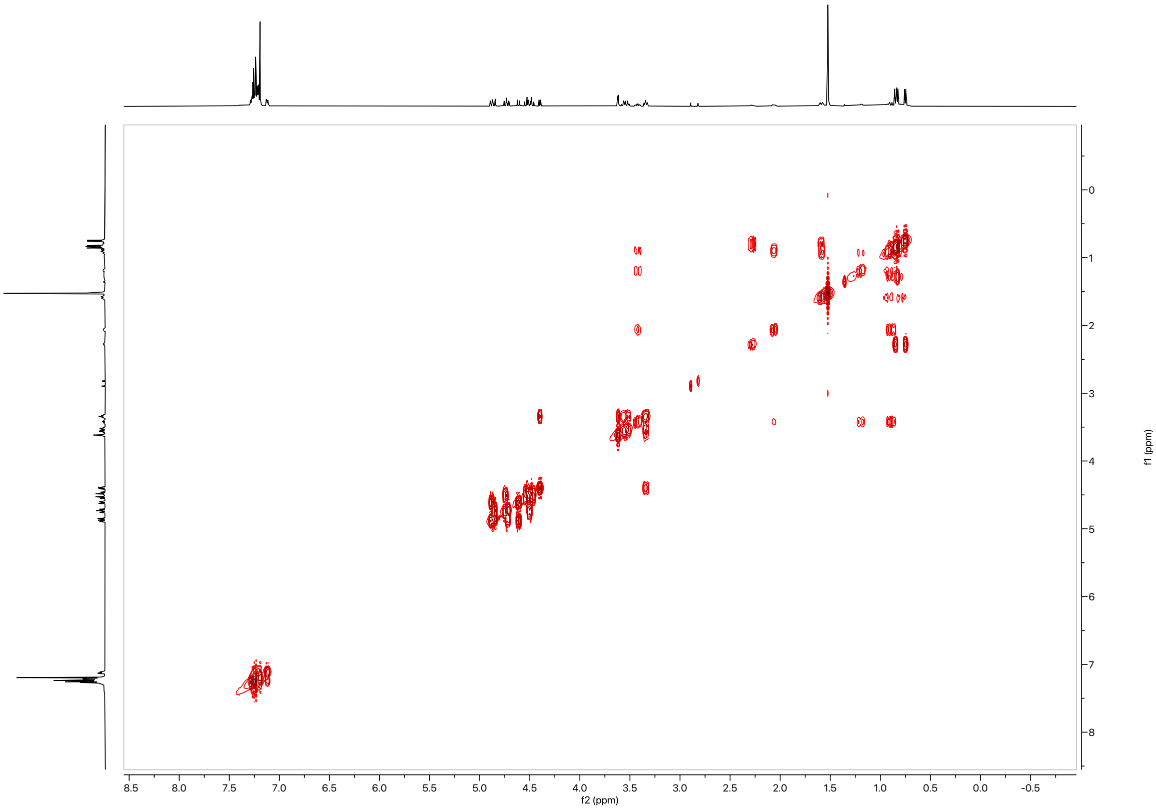

HSQC ^{1}H NMR (500MHz, $CDCl_3$), ^{13}C NMR (126MHz, $CDCl_3$)

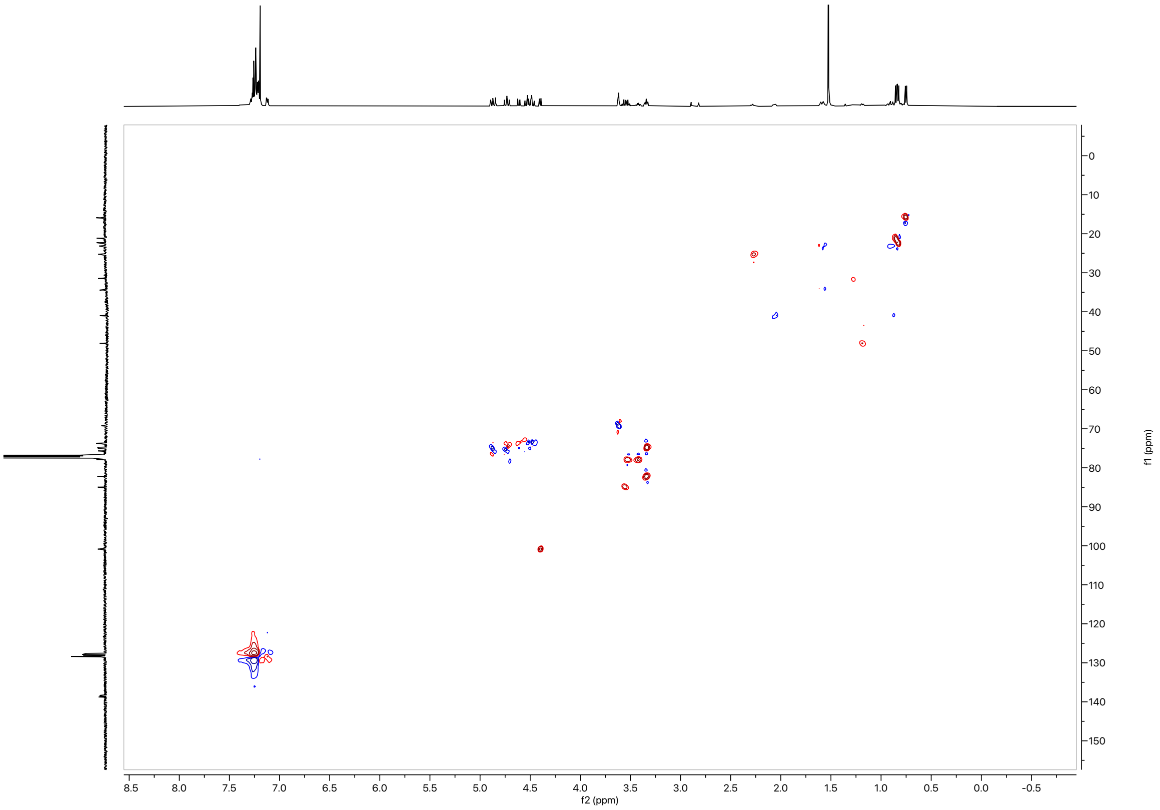

6 References

References

[1] M. M. Nielsen, P. Mała, E. T. Baldursson, and C. M. Pedersen, *Chem. Sci.*, **2019**, *10*(20), 5299–5307.

[2] H. H. Trinderup, S. M. Andersen, M. Heuckendorff, and H. H. Jensen, *Eur. J. Org. Chem.*, **2021**, *2021*(22), 3251–3259.

[3] Agnes Kutt, Toomas Rodima, Jaan Saame, Elin Raamat, Vahur Maemets, Ivari Kaljurand, Ilmar A Koppel, Romute Yu Garlyauskayte, Yurii L Yagupolskii, Lev M Yagupolskii, et al., *J. Org. Chem*, **2011**, *76*(2), 391–395.

[4] Christopher A Reed, *Chem. New Zealand*, **2011**, *75*, 174–179.
